# Comparative genomics of *Nocardia seriolae* reveals recent importation and subsequent widespread dissemination in mariculture farms in the South Central Coast region, Vietnam

**DOI:** 10.1099/mgen.0.000845

**Published:** 2022-07-04

**Authors:** Cuong T. Le, Erin P. Price, Derek S. Sarovich, Thu T. A. Nguyen, Daniel Powell, Hung Vu-Khac, D. İpek Kurtböke, Wayne Knibb, Shih-Chu Chen, Mohammad Katouli

**Affiliations:** ^1^​ Centre for Bioinnovation, University of the Sunshine Coast, Sippy Downs, Queensland, Australia; ^2^​ Institute for Aquaculture, Nha Trang University, Nha Trang, Vietnam; ^3^​ Sunshine Coast Health Institute, Birtinya, Queensland, Australia; ^4^​ Institute for Biotechnology and Environment, Nha Trang University, Nha Trang, Vietnam; ^5^​ Central Vietnam Veterinary Institute, Nha Trang, Vietnam; ^6^​ Department of Veterinary Medicine, College of Veterinary Medicine, National Pingtung University of Science and Technology, Pingtung, Taiwan, ROC; ^7^​ School of Science, Technology and Engineering, University of the Sunshine Coast, Sippy Downs, Queensland, Australia

**Keywords:** *Nocardia seriolae*, trachinotus, nocardiosis, genomics, aquaculture, permit fish, fish infection, infectious disease, fish mortality

## Abstract

Between 2010 and 2015, nocardiosis outbreaks caused by *Nocardia seriolae* affected many permit farms throughout Vietnam, causing mass fish mortalities. To understand the biology, origin and epidemiology of these outbreaks, 20 *N *. *seriolae* strains collected from farms in four provinces in the South Central Coast region of Vietnam, along with two Taiwanese strains, were analysed using genetics and genomics. PFGE identified a single cluster amongst all Vietnamese strains that was distinct from the Taiwanese strains. Like the PFGE findings, phylogenomic and SNP genotyping analyses revealed that all Vietnamese *N. seriolae* strains belonged to a single, unique clade. Strains fell into two subclades that differed by 103 SNPs, with almost no diversity within clades (0–5 SNPs). There was no association between geographical origin and subclade placement, suggesting frequent *N. seriolae* transmission between Vietnamese mariculture facilities during the outbreaks. The Vietnamese strains shared a common ancestor with strains from Japan and China, with the closest strain, UTF1 from Japan, differing by just 220 SNPs from the Vietnamese ancestral node. Draft Vietnamese genomes range from 7.55 to 7.96 Mbp in size, have an average G+C content of 68.2 % and encode 7 602–7958 predicted genes. Several putative virulence factors were identified, including genes associated with host cell adhesion, invasion, intracellular survival, antibiotic and toxic compound resistance, and haemolysin biosynthesis. Our findings provide important new insights into the epidemiology and pathogenicity of *N. seriolae* and will aid future vaccine development and disease management strategies, with the ultimate goal of nocardiosis-free aquaculture.

## Data Summary

Sequence read files (SRX10462095, SRX10462096, SRX10462097, SRX10462093, SRX10462094, SRX10462092, SRX10462098) and the draft genome assemblies of all seven Vietnamese *Nocardia seriolae* strains are available in the National Center for Biotechnology Information (NCBI) Sequence Read Archive under BioProject PRJNA551736 (https://www.ncbi.nlm.nih.gov/bioproject/PRJNA551736 and https://www.ncbi.nlm.nih.gov/genome/browse#!/prokaryotes/14550/).

**Impact Statement**

*
Nocardia seriolae *, the aetiological agent of a lethal granulomatous disease known as fish nocardiosis, has caused high fish mortalities to global aquaculture sectors in recent decades, particularly in Asia and the Americas. This pathogen possesses a highly conserved genome and minimal genetic diversity, which limits the discriminatory power of existing genotyping techniques such as PFGE, leading to insufficient resolution among genetically related strains. To overcome resolution issues using genotyping methods such as PFGE, we employed whole-genome sequencing (WGS) to create highly resolved time-calibrated phylogenies from all available *N. seriolae* genomes (*n*=20), including seven newly sequenced strains we retrieved from Vietnamese fish farms, where nocardiosis outbreaks are increasingly imposing a significant commercial burden. This comprehensive comparative genomic analysis provides the first global phylogenetic analysis of *N. seriolae* strains, allowing the elucidation of the temporal and spatial dynamics of this pathogen, particularly in Vietnam. Using the comparative genomic data, we developed two SNP-based genotyping assays for differentiating Vietnamese from non-Vietnamese strains, and for distinguishing between the two Vietnamese clades, offering an inexpensive tool for rapidly discriminating and tracing the origin of new nocardiosis outbreaks. Our WGS and SNP assays identified the rapid and undetected spread of *N. seriolae* throughout South Central Coast aquaculture facilities, reflecting the need for better surveillance measures for this emerging pathogen. Finally, our genomic analysis also identified multiple virulence factors and antimicrobial resistance genes, which provide valuable information for better understanding the pathogenicity and persistence of this important aquaculture pathogen.

## Introduction

The genus *Trachinotus*, of the family Carangidae, comprises a group of marine, medium-sized, migratory, pelagic finfish that are widely distributed in subtropical and tropical waters worldwide [1, 2]. Many members of the genus, such as *T. carolinus*, *T. blochii, T. ovatus,* and *T. falcatus* are of great economic importance for fisheries and aquaculture sectors in America and Asia due to their high-quality meat, fast growth, high market price, and strong adaptability to a variety of captive environments [3–7]. In Asia, the farming of permit fish, particularly the snub nose permit, *T. falcatus*, has commercially taken place in ponds, raceways, and floating sea cages in both brackish and sea waters. Since 2010, Asian mariculture farms have produced over 2 million tonnes of fish meat, significantly contributing to the food security, poverty alleviation, and economic growth of the region [8]. However, the shortage of quality seed stock and the risk of fish disease outbreaks in several countries are key obstacles and challenges for the sector’s sustainable development.

*T. falcatus* fingerlings were first imported into Vietnam from Taiwan and China in the 2000s and have quickly gained popularity, with permit fish now the third largest group of commercially cultured marine fish after seabass and grouper. However, high mortality rates of *T. falcatus* weighing between 5 and 350 g (6–45 cm in length) emerged in 2010 during an epizootic event that affected sea cage farms in Khánh Hòa province, in the South Central Coast region of Vietnam. Since this initial outbreak, large-scale outbreaks have occurred at several other farming sites in southern and central parts of the country [9, 10]. Infected fish showed clinical signs of nocardiosis such as lethargy, skin blisters, ulcers, and multiple yellowish to whitish nodules affecting both internal and external organs. Based on analyses of 16S rRNA gene sequences and biochemical characteristics, the bacterial pathogen *Nocardia seriolae* was confirmed as the causative agent [10]; however, the origin of *N. seriolae* affecting Vietnamese permit fish farms has not yet been identified.

*N. seriolae* is a Gram-positive, branching, filamentous intracellular bacterium of the family *Nocardiaceae* that was initially described as *N. kampachi* in farmed yellowtail, *Seriola quinqueradiata* [11], following large outbreaks in Mie Prefecture, Japan. An estimated loss of approximately 260 tonnes of cultured yellowtails due to the disease was recorded in 1989 [12]. Nocardiosis has also impacted several other important fish species within the Japanese aquaculture industry such as amberjack (*Seriola dumerili*), Japanese flounder (*Paralichthys olivaceus*), and chub mackerel (*Scomber japonicas*). *N. seriolae* has subsequently been documented in Taiwan, China, Korea, USA, and Mexico, where high mortalities and associated economic losses due to nocardiosis have been reported in freshwater and marine fish species in both cultured and wild populations [13–23]. Despite causing significant economic losses in fish aquaculture worldwide, there are currently no effective measures against nocardiosis.

Five complete and eight draft *N. seriolae* genome sequences were publicly available prior to our study, representing isolates retrieved from Japan, South Korea, and China [24–28]. These genomes have provided important insights into *N. seriolae* epidemiology, transmission, pathogenesis, and infection control strategies; however, isolates from other nocardiosis-prevalent regions such as Taiwan, USA, Mexico, and Vietnam have not yet been examined, leaving major gaps in our understanding of this devastating infectious disease. In the current study, we sequenced the genomes of seven *N. seriolae* isolates isolated from different permit fish farm locations across Vietnam and compared them with the 13 previously genome-sequenced *N. seriolae* isolates, allowing a comparison of isolates spanning a decade in time and from a variety of sources and geographical locations. Using this information, we developed two novel SNP-based PCR assays to rapidly differentiate Vietnam and non-Vietnam strains, and strains representing the two Vietnamese clades. We also characterized potential virulence factors and antimicrobial/toxin resistance determinants to gain insights into pathogenicity and survival mechanisms. Finally, we functionally annotated our *N. seriolae* genomes to determine whether differences in gene content might contribute to physiological variability among isolates.

## Methods

### Bacterial strains

Due to a ban on *N. seriolae* culture importation into Australia, all live culture work was carried out in laboratories at the Institute for Aquaculture, Nha Trang University, Vietnam (for Vietnamese strains) and the Department of Veterinary Medicine, College of Veterinary Medicine, National Pingtung University of Science and Technology, Pingtung, Taiwan (for Taiwanese strains).

**Fig. 1.**
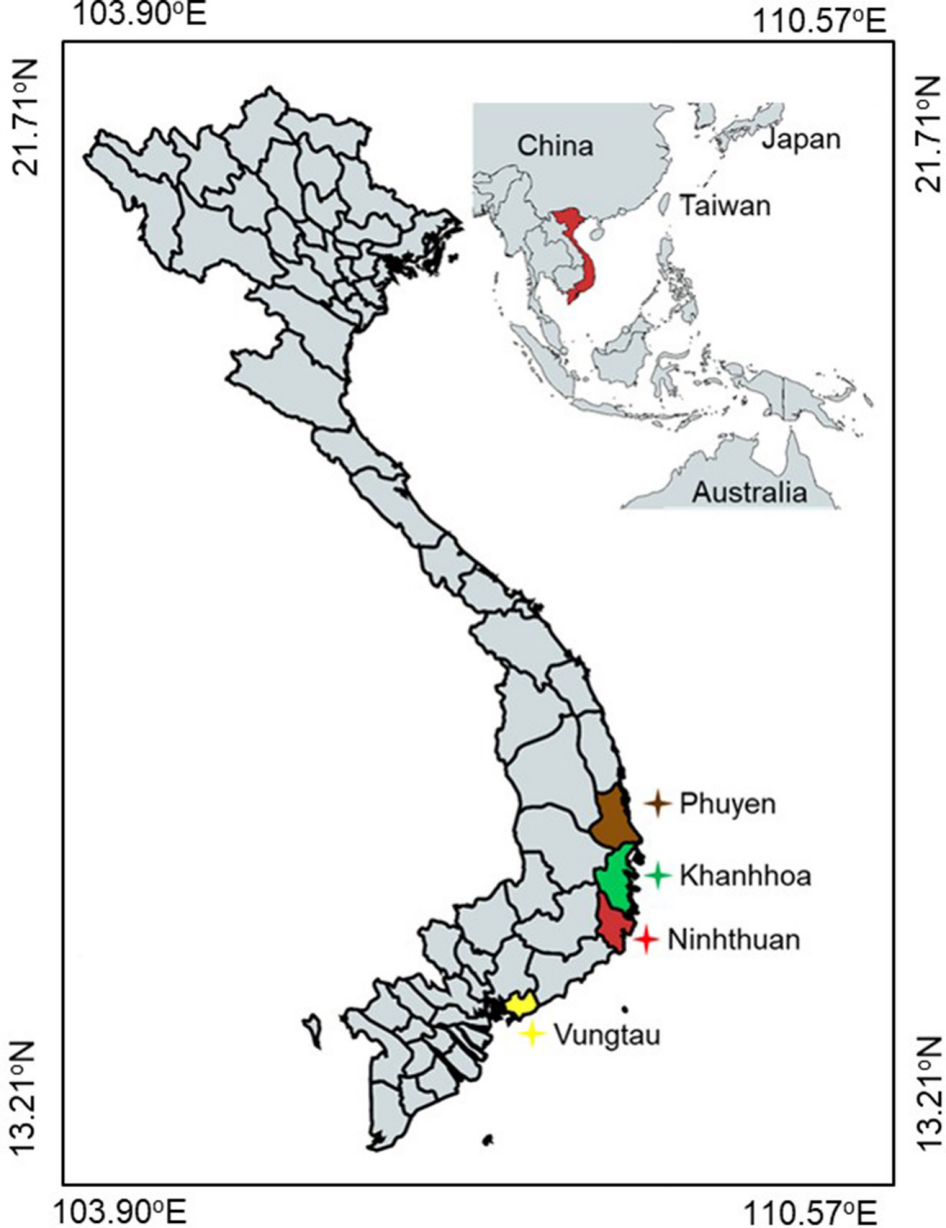
Four Vietnamese provinces where *Nocardia seriolae* isolates were collected from infected permit fish (*Trachinotus falcatus*).

**Fig. 2.**
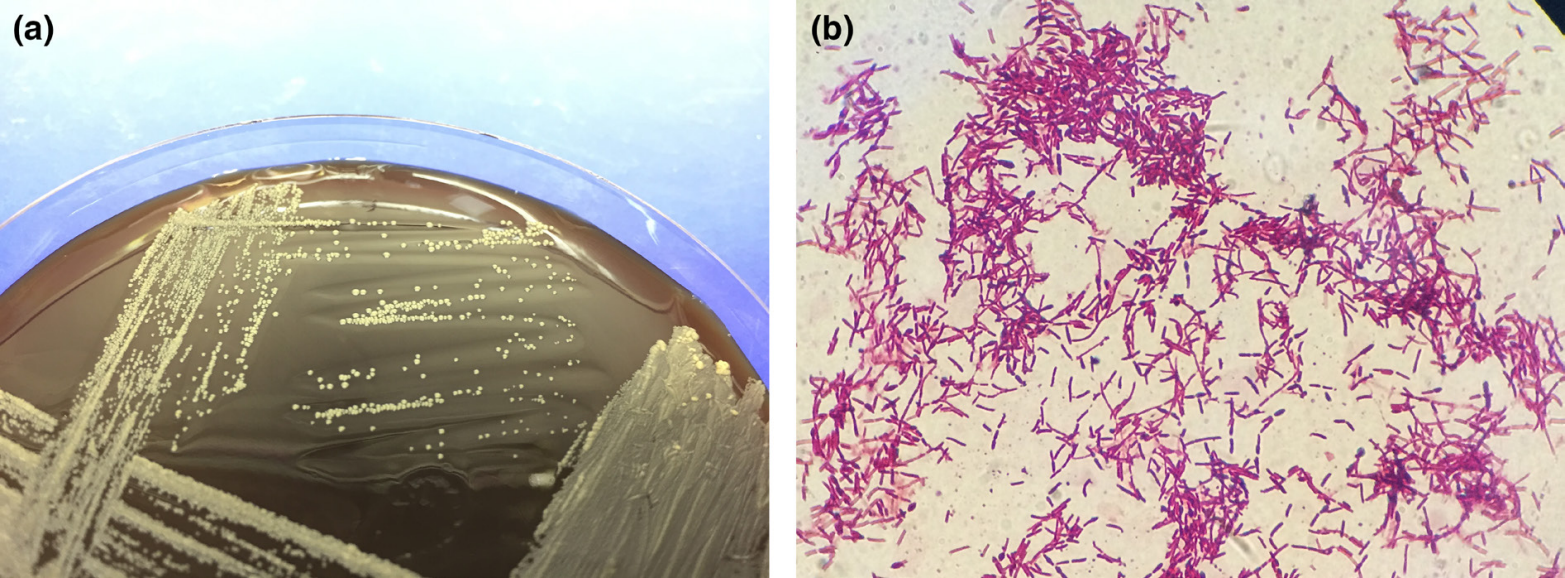
Morphology of *Nocardia seriolae* isolated from Vietnam mariculture farms. (**a**) Chalky white non-haemolytic colonies of *N. seriolae* on sheep blood agar (3-week-old culture); and (**b**) Ziehl–Neelsen-stained *N. seriolae *, showing purple red, filamentous branching bacteria.

Twenty-two *N. seriolae* strains isolated from fish were examined in this study, comprising 20 from Vietnam and two from Taiwan. Vietnamese strains were isolated from cultured permit fish (*T. falcatus*) (31.0–85.8 g) during nocardiosis outbreaks occurring between 2014 and 2015 in four provinces (Phú Yên, Khánh Hòa, Ninh Thuận, and Vũng Tàu) in the South Central Coast region, and the Taiwanese strains were isolated from largemouth bass (*Micropterus salmoides*) and mullet (*Mugil cephalus*) in 2007 (Fig. 1 and Table 1). Isolates were confirmed as *N. seriolae* based on morphological observations, Ziehl-Neelsen staining (Fig. 2), 16S rRNA gene sequencing, and biochemical characteristics. The 20 Vietnamese strains were subject to PFGE analyses, of which seven isolates were selected for whole-genome sequencing (WGS) to enable more detailed genetic analyses. All 22 isolates were tested using our SNP genotyping assays.

**Table 1.** *
Nocardia seriolae* strains collected in this study, their *Ase*I and *Xba*I PFGE profiles, and their SNP genotypes

Country	Strain	Fish species	Host tissue	Origin	Collection date	*Ase*I	*Xba*I	SNP genotype*
Taiwan	96127	*Micropterus salmoides*	Unknown	Taiwan	2007	A1	X1	S1
Taiwan	96994	*Mugil cephalus*	Unknown	Taiwan	2007	A4	X5	S1
Vietnam	KH_11	*Trachinotus falcate*	Muscle	Khánh Hòa, Vietnam	March 2014	NsA2	NsX3	S2C1
Vietnam	KH_14	*Trachinotus falcatus*	Spleen	Khánh Hòa, Vietnam	April 2014	NsA1	NsX1	S2C2
Vietnam	KH_15	*Trachinotus falcatus*	Kidney	Khánh Hòa, Vietnam	May 2014	NsA1	NsX5	S2C1
Vietnam	KH_17	*Trachinotus falcatus*	Spleen	Khánh Hòa, Vietnam	March 2014	NsA1	NsX3	S2C1
Vietnam	KH_21	*Trachinotus falcatus*	Kidney	Khánh Hòa, Vietnam	April 2014	NsA2	NsX3	S2C2
Vietnam	NT_01	*Trachinotus falcatus*	Muscle	Ninh Thuận, Vietnam	April 2014	NsA3	NsX5	S2C2
Vietnam	NT_02	*Trachinotus falcatus*	Spleen	Ninh Thuận, Vietnam	April 2014	NsA3	NsX2	S2C1
Vietnam	NT_03	*Trachinotus falcatus*	Liver	Ninh Thuận, Vietnam	April 2014	NsA5	NsX1	S2C2
Vietnam	NT_50	*Trachinotus falcatus*	Spleen	Ninh Thuận, Vietnam	April 2014	NsA2	NsX3	S2C2
Vietnam	PY_22	*Trachinotus falcatus*	Spleen	Phú Yên, Vietnam	April 2014	NsA4	NsX1	S2C1
Vietnam	PY_23	*Trachinotus falcatus*	Muscle	Phú Yên, Vietnam	April 2014	NsA9	NsX1	S2C1
Vietnam	PY_30	*Trachinotus falcatus*	Liver	Phú Yên, Vietnam	April 2014	NsA8	NsX1	S2C2
Vietnam	PY_31	*Trachinotus falcatus*	Bone	Phú Yên, Vietnam	April 2014	NsA10	NsX4	S2C1
Vietnam	PY_35	*Trachinotus falcatus*	Spleen	Phú Yên, Vietnam	April 2014	NsA7	NsX1	S2C2
Vietnam	PY_37	*Trachinotus falcatus*	Spleen	Phú Yên, Vietnam	April 2014	NsA6	NsX1	S2C2
Vietnam	PY_39	*Trachinotus falcatus*	Spleen	Phú Yên, Vietnam	April 2014	NsA7	NsX1	S2C2
Vietnam	PY_40	*Trachinotus falcatus*	Kidney	Phú Yên, Vietnam	April 2014	NsA6	NsX1	S2C1
Vietnam	VT_45	*Trachinotus falcatus*	Spleen	Vũng Tàu, Vietnam	June 2015	NsA10	NsX3	S2C1
Vietnam	VT_61	*Trachinotus falcatus*	Spleen	Vũng Tàu, Vietnam	June 2015	NsA11	NsX1	S2C1
Vietnam	VT_62	*Trachinotus falcatus*	Liver	Vũng Tàu, Vietnam	June 2015	NsA12	NsX1	S2C2

*S1, non-Vietnamese SNP genotype; S2, Vietnamese SNP genotype; C1, Vietnam Clade 1; C2, Vietnam Clade 2.

Isolates were preserved in Brain Heart Infusion (BHI; Difco) broth mixed with 25 % (v/v) glycerol and stored at −80 °C. For culturing, strains were grown in BHI broth at 28 °C for 5 days, with orbital shaking at 150 r.p.m. For DNA extraction, 0.3 ml of bacterial cells were pelleted at 6000 ***g*** at 4 °C for 5 min and washed twice with 1× sterile PBS. To test for a haemolytic reaction, *N. seriolae* colonies grown in BHI broth were streaked onto 5 % (v/v) sheep blood agar and incubated at 28 °C for 3 weeks (Fig. 2a).

### PFGE typing

PFGE was performed using 50 U *Xba*I or *Ase*I (New England BioLabs) as previously described [20]. The type strain, *N. seriolae* BCRC 13745 (JCM 3360; isolated from the spleen of farmed yellowtail in Nagasaki Prefecture, Japan, *ca.* 1974), was included for comparative purposes. Gels of DNA fragments were analysed using GelCompar II software version 6.5 (Applied Maths). Gel bands were automatically assigned by the software and were checked and corrected manually. Only clearly resolved bands were considered for further analysis. A dendrogram was constructed using an unweighted pair group method with arithmetic mean (UPGMA) approach and the Dice similarity coefficient, with band optimization and band position tolerances of 1.0 %. Isolates that showed similarity between the banding profiles of ≥80 % (fewer than six bands of difference) were defined as indistinguishable or clonally related, whereas patterns with <80 % similarity (six or more bands of difference) represented different clusters of unrelated strains [29, 30].

### DNA extraction

Total genomic DNA of bacterial isolates was extracted using the Wizard Genomic DNA Purification Kit (Promega) as per the manufacturer’s instructions. DNA was checked for sterility and shipped to the University of the Sunshine Coast, Queensland, Australia. The quantity and purity of extracted DNA were assessed using a NanoDrop 2000 (Thermo Scientific) and 1 % gel electrophoresis. DNA for Illumina WGS was submitted on dry ice to the Australian Genome Research Facility (AGRF; North Melbourne, VIC, Australia).

### WGS and comparative genomic analyses

NextEra DNA Flex Illumina libraries for seven Vietnamese *N. seriolae* isolates were sequenced in four lanes of a single flowcell on the NextSeq 500 platform (Illumina), to produce 150 bp paired reads at an average depth of ~ 390× (range: 326–433×). Raw read quality was assessed with FastQC v0.11.5 (http://www.bioinformatics.babraham.ac.uk/projects/fastqc/). These seven genomes are available on the Sequence Read Archive database under BioProject PRJNA551736. Thirteen publicly available genome assemblies (strains EM150506, CK-14008, HSY-NS01, HSY-NS02, MH196537, N-2927, NBRC 15557, NK201610020, SY-24, U-1, UTF1, ZJ0503 and TL20, corresponding to GenBank assembly references ASM186585v1, ASM188553v1, ASM301359v1, ASM366707v1, ASM1411730v1, ASM58371v2, ASM799071v1, ASM1520982v1, ASM209393v1, ASM119293v1, ASM235603v1, ASM76316v1 and ASM1822368v1, respectively) were converted to simulated Illumina reads using ART v2016.06.05 [31] prior to analysis. EM150506, the largest complete *N. seriolae* genome (GenBank accession number CP017839.1) [28], was used as the reference sequence for read mapping and gene annotation. Biallelic, orthologous SNPs from the 20 *N *. *seriolae* genomes were identified using the default settings of SPANDx v4.0.1 [32], which integrates the Burrows–Wheeler Aligner [33], Sequence Alignment/Map (SAM) tools [34], BEDTools [35], VCFtools [36], Picard Tools (http://broadinstitute.github.io/picard) and Genome Analysis Toolkit [37] into a single pipeline.

**Fig. 3.**
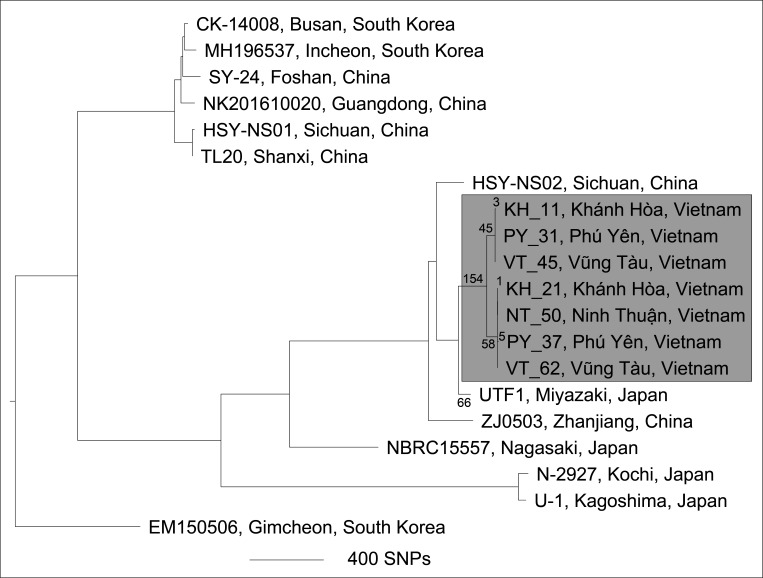
Rooted maximum-parsimony phylogenomic analysis of seven Vietnamese (KH_11, KH_21, NT_50, PY_31, PY_37, VT_62 and VT_45; grey box) and 13 non-Vietnamese *Nocardia seriolae* genomes using EM150506 (Fig. S2) as an outgroup. A total of 7343 high-confidence biallelic, orthologous, core-genome SNPs were used to reconstruct the phylogeny. Branch lengths within the Vietnamese clade are labelled and refer to the number of SNPs along each branch. Consistency index=0.998.

We performed a hierarchical rooted phylogenetic approach to identify the appropriate root for our *N. seriolae *-only phylogeny (Fig. 3). First, we identified the nearest genetic neighbour to *N. seriolae* via a SPANDx phylogenomic comparison of 134 *Nocardia* species genomes belonging to 78 assigned species and 10 unassigned species (Fig. S1, available in the online version of this article). Next, we reconstructed a rooted phylogeny using the closest relative, *N . concava* NBRC 100430 (RefSeq accession: GCF_000308815.1) (Fig. S2), to determine the most ancestral *N. seriolae* strain for phylogenetic rooting.

Using the SPANDx SNP matrices, maximum-parsimony phylogenomic trees were reconstructed by Phylogenetic Analysis Using Parsimony (PAUP*) v4.0a168 software [38], with trees visualized using FigTree v1.4.0 (http://tree.bio.ed.ac.uk/software/figtree/). For the *N. seriolae *-only phylogeny, variant annotation was also carried out using SnpEff [39] (Data S1). To determine similarity among *N. seriolae* genomes, and to check for potential rearrangements, contigs in all genome assemblies were oriented and arranged against the reference genome using MAUVE v2.3.1 [40]. blast Ring Image Generator (BRIG) [41] was subsequently used to visualize genome relatedness and structural variation. Finally, temporal analysis was performed with BEAST v1.10.4 [42] using the approach outlined by Holt and colleagues [43, 44].

### SNP genotyping

The SPANDx SNP matrix was used to identify SNPs that: (i) distinguished Vietnamese from non-Vietnamese *N. seriolae* strains (220 SNPs; SNP1 assay), and (ii) differentiated the two Vietnamese clades (103 SNPs; SNP2 assay). We selected SNPs at positions 60409 and 587171 in EM150506 for SNP1 and SNP2 assay design, respectively (Data S1). SYBR Green-based mismatch amplification mutation assay (SYBR-MAMA) real-time PCRs were developed to permit rapid genotyping of all strains from this study against these two SNPs. SYBR-MAMA, also known as allele-specific PCR or amplification-refractory mutation system, exploits the differential 3′ amplification efficiency of *Taq* polymerase in real time via allele-specific primers targeting each SNP allele at their ultimate 3′-end [45]. SYBR-MAMA has been used for SNP genotyping in many bacteria [46, 47] due to its low cost and simplicity . Each SNP assay consisted of one common primer and two allele-specific primers, matching either the non-Viet allele or the Viet allele for the SNP1 assay, and the Viet Clade 1 allele or Viet Clade 2 allele for the SNP2 assay (Table 2). The same destabilizing mismatch (A for SNP1 and G for SNP2) was incorporated at the penultimate (−2) 3′ base of both allele-specific primers to increase allele specificity [48]. Cycles-to-threshold (*C*_T_) values for each allele-specific reaction were used to determine the SNP genotype for each strain via a change in *C*_T_ value (Δ*C*_T_).

**Table 2.** Primer sequences of SYBR-MAMAs designed in this study for the differentiation of Vietnamese *Nocardia seriolae* strains

SNP assay and target	SNP position*	Variation (allele base)	Primer name	Primer sequence†
SNP1 (Vietnam vs. non-Vietnam strains)	60409	C/T	CtS1_nonViet_For	CAAACCGGCTGGATATCGa**C**
			CtS1_Viet_For	CAAACCGGCTGGATATCGa**T**
			SNP1_Rev	CACGCCGACGCTAGTACCTG
SNP2 (Vietnam subclades 1 vs. 2)	587171	A/C	CtS2_Clade1_Rev	CATACCGACTTCCAGGTGTGg**T**
			CtS2_Clade2_Rev	ACCGACTTCCAGGTGTGg**G**
			SNP2_For	AGCCCATTAGCAGTCGTGTGA

*SNP position as per *N. seriolae* EM150506 [28] (GenBank reference CP017839.1).

†Single 3′ penultimate mismatch bases are shown in lowercase; SNP-specific nucleotides are indicated in bold.

SYBR-MAMA, SYBR Green-based mismatch amplification mutation assay.;

To validate SNP genotypes for our newly developed assays, we first established the reference Δ*C*_T_ values for each assay by running against the two Taiwanese and seven genome-sequenced Vietnamese strains. Assays were then tested against the 13 remaining Vietnamese isolates to determine their genotypes. For each PCR run, control DNA samples representing the matching and non-matching allele genotypes were used as positive controls, and at least two no-template controls were included.

SYBR-MAMAs contained 1 µl of target DNA template at ~1 ng µl^–1^, 0.2 µM allele-specific primer, 0.2 µM common primer (Macrogen), 1× Platinum SYBR Green qPCR SuperMix-UDG (cat. no. 11733038; Thermo Fisher Scientific) and RNase/DNase-free PCR-grade water (Cat No. 10977015; Thermo Fisher Scientific), to a total reaction volume of 5 µl. Thermocycling conditions comprised an initial 2 min denaturation at 95 °C, followed by 45 cycles of 95 °C for 15 s and 60 °C for 15 s. All samples were run in duplicate.

### Genome assembly and annotation

Assemblies of the seven Vietnamese *N. seriolae* genomes were constructed from the quality-filtered sequence data using the Microbial Genome Assembly Pipeline (MGAP) v1.1 (https://github.com/dsarov/MGAP---Microbial-Genome-Assembler-Pipeline) and EM150506 (GenBank reference CP017839.1) as the scaffolding reference. MGAP wraps Trimmomatic [49], Velvet [50], VelvetOptimiser (https://github.com/tseemann/VelvetOptimiser), ABACAS [51], IMAGE [52], SSPACE [53, 54], GapFiller [55, 56] and Pilon [57] into a single tool. Assemblies were primarily annotated using the Rapid Annotations using Subsystems Technology (RAST) server v2.0 with SEED data with default features (RAST annotation scheme: RASTtk, automatically fix errors, fix frameshifts, build metabolic model, backfill gaps, turn on debug, verbose level: 0, and disable replication: yes). RAST was also used to group genes into functional subsystems (akin to Clusters of Orthologous Groups). Annotated genomes were then compared with results provided by Prokka v1.8 [58]. In cases where aberrant results arose between the two tools, the functional prediction of RAST was checked and manually corrected by using BLASTP to search for similar proteins in the UniProtKB database (http://www.uniprot.org/blast/). The clustered regularly interspaced short palindromic repeat (CRISPR)-Cas region finder program (https://crisprcas.i2bc.paris-saclay.fr) was used to identify regular repeats and the intervening spacer sequences [59]. The assembled genomes for all Vietnamese strains are available from NCBI under BioProject PRJNA551736 (Table 3).

### Virulence and antimicrobial resistance profile determination

The identification of antimicrobial resistance- and virulence-related genes among the Vietnamese *N. seriolae* genomes were performed using RAST and the Virulence Factor Database (VFDB), Victors and PATRIC Virulence Factor (VF) databases available on the Pathosystems Resource Integration Center (PATRIC) [60, 61]. In addition, homologues of experimentally verified pathogenicity determinants within other members of the genus *Nocardia* were searched for in the *N. seriolae* genomes.

## Results

### PFGE genotypes

**Fig. 4.**
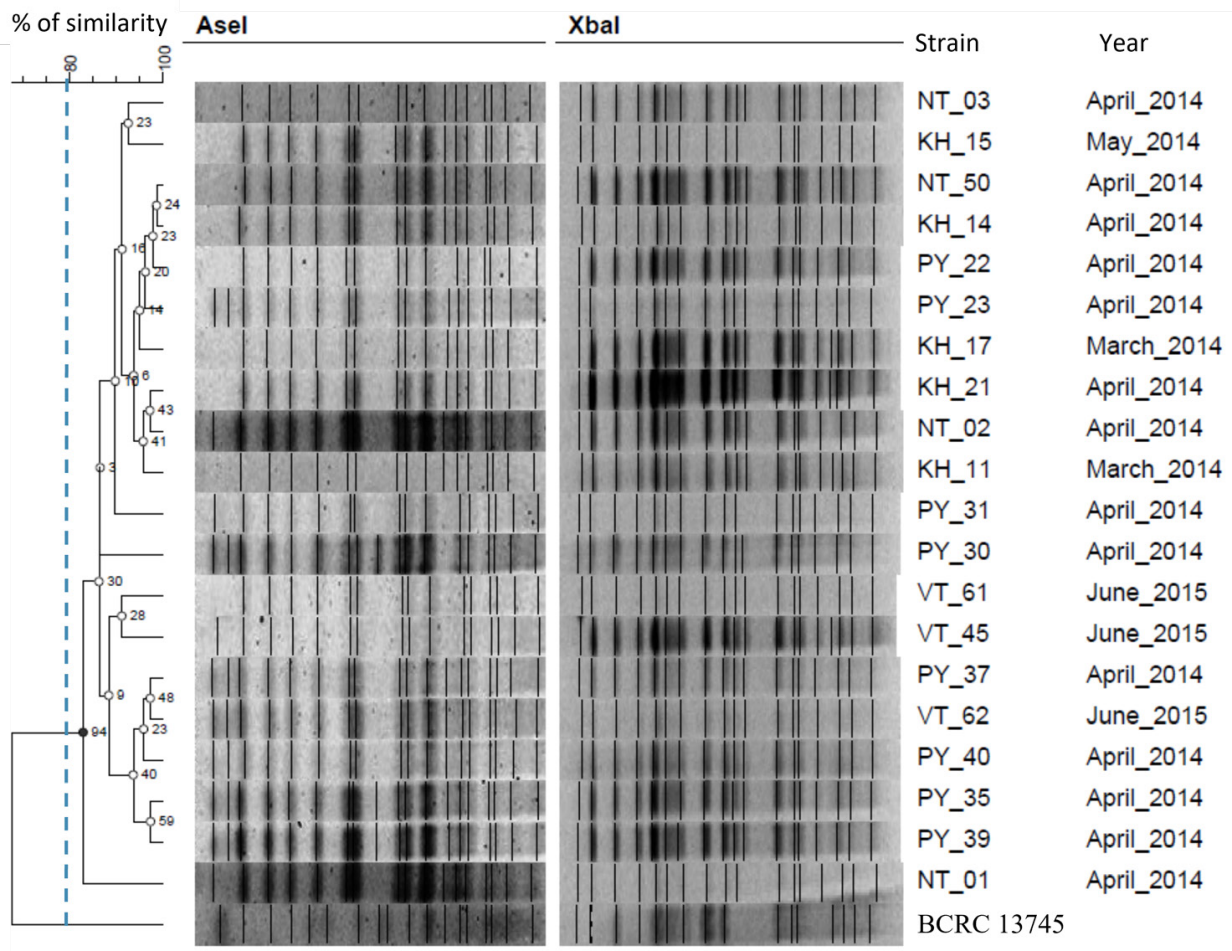
PFGE dendrogram of *Ase*I- and *Xba*I-digested genomic DNA from 20 representative *Nocardia seriolae* strains collected in four Vietnamese provinces. A type strain, BCRC 13745 (Japan), was included for comparison. Cluster analysis of genetic distances was performed using the Dice coefficient and UPGMA method (tolerance and optimization 1%). Two pulsotypes were identified based on an 80 % similarity cut-off. Numbers at tree nodes indicate the percentage of replicate trees in which the same clusters were found after 1000 bootstrap replicates.

Twenty *N. seriolae* isolates from four Vietnamese coastal provinces (Fig. 1) were subjected to *Xba*I and *Ase*I digestion to determine isolate relatedness across provinces. Restriction fragment sizes ranged from 40 kb to 1.1 Mbp. PFGE with *Xba*I alone resulted in between 19 and 21 restriction fragments among the Vietnamese strains; similarly, between 16 and 20 fragments were identified using *Ase*I. Seven distinct patterns (labelled as pulsotypes NsX1–NsX7) were present using *Xba*I-digested DNA fragments, and ten patterns (labelled as pulsotypes NsA1–NsA10) for *Ase*I. Using the ≥80 % similarity cut-off and ‘fewer than six bands of difference’ Tenover criteria, only one cluster was identified for each enzyme [29, 30]. Even when combining data from both enzymes, the 20 Vietnamese isolates were still closely related, irrespective of their geographical origin, as shown by their categorization into a single cluster that was distinct from the Japanese type strain (Fig. 4).

### Phylogenomic analysis

Based on the PFGE results, seven geographically diverse Vietnamese isolates were Illumina-sequenced, resulting in high-coverage draft genomes (Table 3). These genomic data were generated to address two questions: (i) whether comparative genomics, as with PFGE, would reveal minimal genetic diversity among the Vietnamese *N. seriolae* strains, and (ii) whether phylogenomic analysis could identify a potential origin for nocardiosis in Vietnamese aquaculture facilities. The seven Vietnamese genomes generated in this study, plus the sequences of 13 publicly available *N. seriolae* strains (all from other Asian countries), were compared to identify phylogenetically informative SNPs. A total of 8206 SNPs were identified; 7517 (91.6 %) were located in coding regions and comprised 126 nonsense, 5163 missense and 1531 silent variants. Of the 8206 SNPs, 7275 high-confidence, orthologous, core genome, biallelic SNPs were identified among the 20 *N *. *seriolae* strains; these SNPs were used for phylogenomic reconstruction.

The phylogenomic dendrogram revealed five distinct strain clusters (Fig. 3). As with PFGE, the seven Vietnamese isolates were highly clonal, with all strains clustering into a single unique ‘Vietnamese’ clade. Within this clade were two subclades that differed by 103 SNPs. These subclade SNPs were well distributed across the genome, with no evidence of SNP clusters due to recombination. The phylogenomic analysis also suggested that *N. seriolae* undergoes very little, if any, recombination, as demonstrated by a very high consistency index of 0.997; in other words, homoplastic SNP characters, which are more common following recombination events [62], were essentially absent. Within the two Vietnamese subclades, isolates were virtually identical (0–5 SNPs), indicating limited genomic alterations among these lineages (Fig. 3). Notably, there was no link between geographical region and subclade placement, with strains from Phú Yên, Khánh Hòa and Vũng Tàu falling into both Vietnamese subclades, indicating frequent *N. seriolae* transmission events between regions. The most recent common ancestor of the Vietnamese strains differed by 220 SNPs from the next closest known strain, UTF1, which was isolated from cultured yellowtail that succumbed to nocardiosis in 2008 in Miyazaki Prefecture, Japan [27].

BEAST analysis (Fig. S3) showed that the most recent common ancestor (MRCA) for the Vietnamese and Japanese *N. seriolae* strains occurred in ~1998 [95 % highest posterior density (HPD): 1997–1999], and all Vietnam *N. seriolae* strains shared an MRCA in 2001 (95 % HPD: 1999–2003).

### SNP genotyping

SYBR-MAMAs demonstrated clear distinction of SNP genotypes. For the SNP1 assay, the two Taiwanese strains amplified the non-Viet allele earlier than the Viet allele (Δ*C*_T_ range: 2.8–5.5); in contrast, all Vietnamese strains amplified the Viet allele earlier than the non-Viet allele (Δ*C*_T_ range: 6.0–9.3). For the SNP2 assay, 10 Vietnamese strains belonging to Clade 1 amplified the Clade 1 allele earlier than the Clade 2 allele (Δ*C*_T_ range: 9.9–13.4), whereas 10 Clade 2 strains amplified the Clade 2 allele earlier (Δ*C*_T_ range: 4.5–8.1) (Table 1). No amplification was observed for the no-template controls.

### Genome assembly and functional annotation

**Fig. 5.**
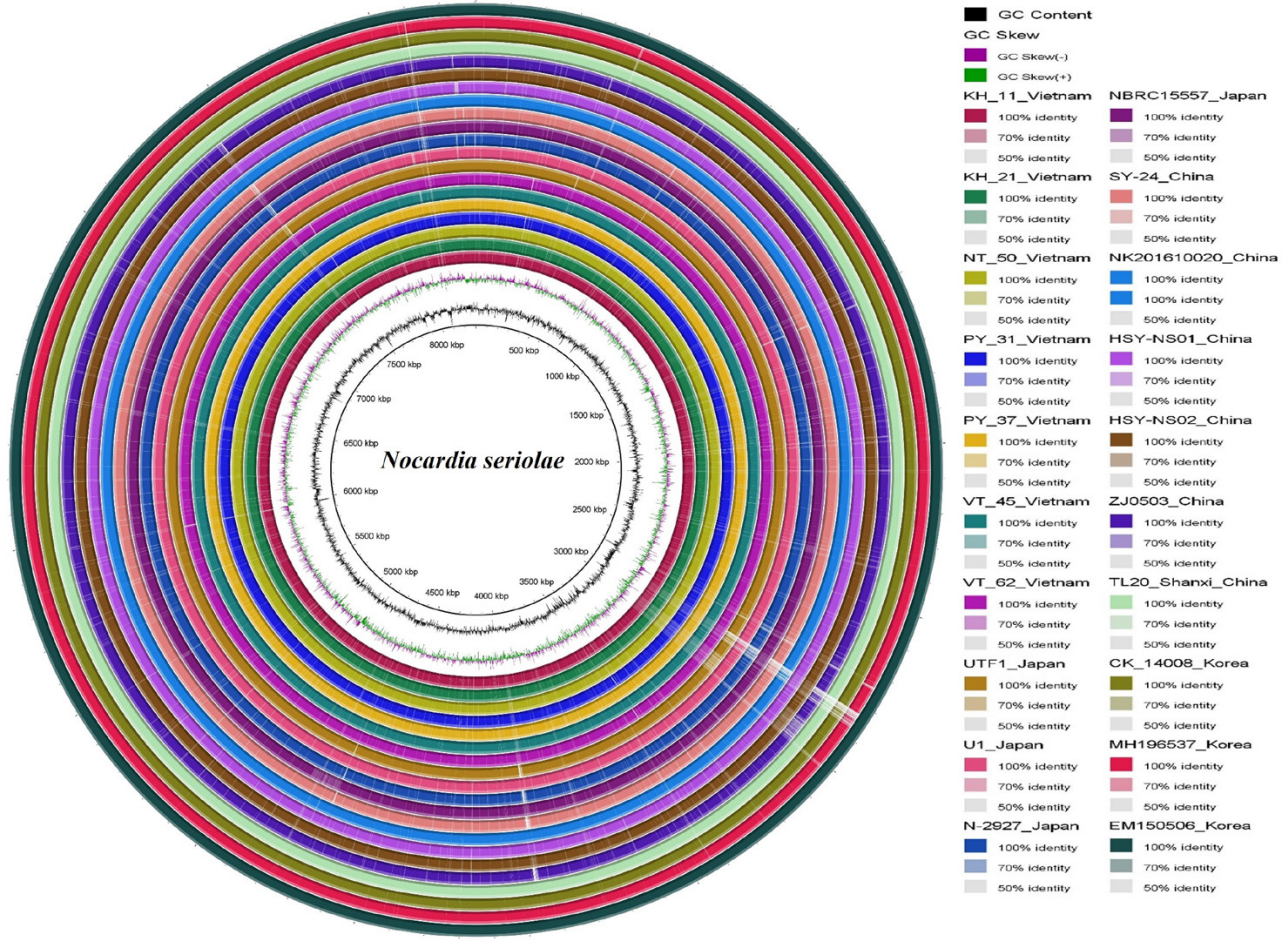
Whole-genome comparison of *Nocardia seriolae* strains from Vietnam and other Asian countries against the EM150506 (South Korean) reference genome using the circular BLASTn alignment in blast Ring Image Generator [39]. The innermost circle shows genome scale (bp), the black irregular ring represents %GC content, and the irregular purple/green ring represents %GC skew. Outer colour rings (innermost first) represent Vietnamese strains (KH_11, KH_21, NT_50, PY_31, PY_37, VT_45, VT_62) and 13 strains from Japan, China, and South Korea. The outermost circle (dark green) represents the EM150506 reference genome.

To gain deeper insights into the seven Vietnamese *N. seriolae* genomes, we conducted a comparative analysis of genome assembly metrics and gene function. The Vietnamese genomes possess 6937 core genes and encode 1–6 rRNA genes and 49–63 transfer RNA genes. Total assembly length ranged from 7.55 to 7.96 Mbp, smaller than the closed genomes EM150506 (8.30 Mbp), MH196537 (8.26 Mbp), UTF1 (8.12 Mbp), and draft genomes reported for CK-14008 (8.37 Mbp) and NK201610020 (8.31 Mbp), but similar to other draft genomes of this species (range: 7.61 to 7.91 Mbp). GC content (68.2–68.3 %) was comparable to previously sequenced *N. seriolae* genomes (Table 3). Multiple genome alignment of all strains against EM150506 using BRIG showed a high degree of homology (Fig. 5), demonstrating high conservation among *N. seriolae* genomes. There were four main non-homologous regions (positions 2 700 000–3 100 000, 3 900 000–4 100 000, 7 500 000–7 600 000 and 8 000 000–8 200 000 bp) that were present in the reference genome but absent in all other genomes; these regions may indeed be absent or may simply reflect differences in assembly quality [5]. Most genes at these loci were classified as hypothetical proteins, mobile element proteins and repeat regions; the remaining loci are mainly genes involved in membrane transport, biosynthesis, metabolism and transcription (Data S2).

**Table 3.** Genetic and genomic features of the Vietnamese *Nocardia seriolae* strains compared with the South Korean EM150506 strain according to RAST

Strains/feature	Country	Genome size (Mbp)	Level of completion	Sequencing platform	Sequencing depth	GC%	L50 (bp)	Total no. of proteins	No. of RNAs	No. of hypothetical proteins	No. of proteins with function prediction	No. of proteins assigned to subsystem	NCBI accession no.
KH_11	Vietnam	7.66	Draft	NextSeq 500	340×	68.3	90	7655	58	3560	4465	2055	WMKE00000000.1
KH_21	Vietnam	7.72	Draft	NextSeq 500	424×	68.2	58	7657	66	3597	4428	2033	WMKF00000000.1
NT_50	Vietnam	7.96	Draft	NextSeq 500	395×	68.2	86	7640	66	3571	4437	2063	WMKG00000000.1
PY_31	Vietnam	7.68	Draft	NextSeq 500	408×	68.3	62	7602	62	3212	4818	2220	WMKC00000000.1
PY_37	Vietnam	7.55	Draft	NextSeq 500	326×	68.3	126	7707	51	3549	4525	2087	WMKD00000000.1
VT_45	Vietnam	7.94	Draft	NextSeq 500	404×	68.2	70	7958	67	3609	4718	2054	WMKB00000000.1
VT_62	Vietnam	7.7	Draft	NextSeq 500	433×	68.3	62	7643	63	3580	4428	2052	WMKH00000000.1
UTF1	Japan	8.12	Complete	PacBio	133×	68.1	1	7890	75	3572	4683	2219	AP017900.1
U-1	Japan	7.77	Draft	Roche 454; MiSeq	179×	68.3	56	7757	69	3645	4497	2291	BBYQ00000000.1
N-2927	Japan	7.76	Draft	Roche 454	160×	68.3	54	7627	66	3225	4841	2245	BAWD00000000.2
NBRC15557	Japan	7.61	Draft	Roche 454; HiSeq 1000	112×	68.3	51	*7527*	64	3190	4768	2211	NZ_BJWY01000001.1
SY-24	China	7.89	Draft	MiSeq	100×	68.2	52	7632	66	3227	4845	2230	MVAC00000000.1
NK201610020	China	8.31	Complete	HiSeq; PacBio	100×	68.1	1	8133	78	3398	5185	2306	NZ_CP063662.1
HSY-NS01	China	7.91	Draft	HiSeq	126×	68.2	50	7947	70	3727	4605	2133	PXZE00000000.1
HSY-NS02	China	7.76	Draft	HiSeq	110×	68.2	51	7801	69	3301	4932	2225	RCNK00000000.1
ZJ0503	China	7.71	Draft	MiSeq	100×	68.3	50	7579	66	3212	4798	2204	JNCT00000000.1
TL20	China	8.3	Complete	PacBio	200×	68.1	1	7710	66	3212	4798	2204	GCA_018223685.1
CK-14008	Korea	8.37	Draft	PacBio	139×	68.1	1	8212	78	3422	5244	2347	MOYO00000000.1
MH196537	Korea	8.26	Complete	PacBio	118×	68.1	1	8074	78	3368	5155	2296	CP059737.1
EM150506	Korea	8.3	Complete	PacBio	156×	68.1	1	8068	77	3338	5175	2277	CP017839.1

RAST predicted between 7602 and 7958 coding DNA sequences in the Vietnamese *N. seriolae* genomes, of which 45.8 % (range: 42.2–47.0 %) are of unknown function (‘hypothetical proteins’). Of the 59.1 % (range: 57.8–63.4 %) coding DNA sequences with RAST function predictions, 45.8 % (range: 43.5–50.9 %) grouped into 308–330 functional subsystems belonging to 24 protein family categories. These predictions are similar to the previously reported *N. seriolae* genomes (Table 4). Little difference was found in the number of genes in family categories among Vietnamese vs. non-Vietnamese strains (Table 4). No plasmids were identified in any of the Vietnamese genomes, consistent with most *N. seriolae* genomes lacking plasmids; the only exception is CK-14008 from South Korea, which potentially harbours two plasmids [28].

**Table 4.** Number of genes for each *Nocardia seriolae* strain associated with the 24 general Clusters of Othologous Groups functional categories predicted by RAST

Functional category	KH_11	KH_21	NT_50	PY_31	PY_37	VT_45	VT_62	UTF1	U-1	N-2927	NBRC 15557	SY-24	NK 201610020	HSY-NS01	HSY-NS02	ZJ0503	TL20	CK-14008	MH196537	EM150506
Cofactors, Vitamins, Prosthetic Groups, Pigments	198	195	196	207	206	195	194	204	211	208	209	204	210	199	205	202	198	212	209	208
Cell Wall and Capsule	32	31	31	36	31	31	31	36	36	36	36	34	36	31	36	36	31	38	36	36
Virulence, Disease and Defence	50	47	48	56	50	53	47	55	58	59	55	57	58	49	55	55	49	60	59	62
Potassium metabolism	10	10	10	11	10	11	10	11	10	11	10	11	10	10	10	10	10	11	12	10
Miscellaneous	30	30	30	33	33	30	30	33	32	32	32	32	32	29	33	33	29	32	32	31
Phages, Prophages, Transposable elements, Plasmids	7	5	5	13	6	5	7	10	16	12	8	15	16	11	12	11	9	17	16	10
Membrane Transport	31	31	31	35	31	31	31	35	37	37	37	37	37	32	35	35	32	37	37	36
Iron acquisition and metabolism	14	14	14	15	14	14	14	15	14	15	15	15	15	14	15	15	14	15	15	15
RNA metabolism	56	58	58	59	56	60	58	61	58	59	57	58	62	58	59	56	60	63	62	62
Nucleosides and Nucleotides	96	96	96	107	98	95	97	101	100	100	106	99	101	95	106	101	95	103	101	100
Protein Metabolism	219	224	225	228	212	229	221	242	238	234	233	233	246	229	236	230	237	248	246	248
Regulation and Cell signalling	23	23	23	26	23	23	23	26	26	26	26	26	26	23	27	26	24	26	26	26
Secondary metabolism	4	4	4	4	4	4	4	4	4	4	4	4	4	4	4	4	4	4	4	4
DNA metabolism	100	99	100	100	105	101	99	102	101	101	100	102	101	99	101	102	101	105	101	100
Fatty Acids, Lipids and Isoprenoids	226	219	243	274	229	223	239	272	310	275	273	273	311	280	273	270	281	319	308	304
Nitrogen Metabolism	32	32	32	35	32	32	32	35	36	36	28	36	35	33	35	35	33	35	36	36
Dormancy and Sporulation	1	1	1	1	1	1	1	1	1	1	1	1	1	1	1	1	1	1	1	1
Respiration	101	100	100	104	107	103	99	103	104	103	77	102	103	99	104	104	98	104	104	104
Stress Response	56	54	55	59	55	56	54	58	58	61	58	61	58	54	60	60	52	59	57	57
Metabolism of Aromatic Compounds	26	26	26	32	27	27	27	32	33	32	32	33	33	26	33	33	27	32	33	34
Amino Acids and Derivatives	365	369	369	391	371	365	367	394	411	406	414	404	415	387	392	392	385	417	412	399
Sulphur Metabolism	14	13	14	13	16	13	14	12	12	14	12	14	13	14	13	14	13	13	13	13
Phosphorus Metabolism	27	27	26	27	27	27	27	27	27	27	27	27	27	27	27	27	27	27	27	27
Carbohydrates	337	325	326	354	343	325	326	350	358	356	361	352	356	329	353	352	329	369	349	354

A typical CRISPR-Cas system contains both a CRISPR array of repeat and spacer units, and associated *cas* genes; however, many systems are devoid of one of these components. These atypical CRISPR configurations are known as ‘orphan’ or ‘isolated’ CRISPR arrays and *cas* loci depending on which component is lacking. Between three and six CRISPR arrays were found in the Vietnamese strains, with lengths varying from 73 to 114 bp. Each array is made up of two direct repeats and one spacer without nearby Cas (CRISPR-associated) genes (Data S3). Notably, the same CRISPR array structure was found in all 20 *N *. *seriolae* genomes.

### Virulence and antimicrobial/toxin resistance profiles

To explore the pathogenic potential of the Vietnamese *N. seriolae* strains, we assessed their virulence and antimicrobial/toxin resistance gene content in comparison to non-Vietnamese genomes. The RAST, VFDB, Victors and VF databases found between 182 and 202 genes that encode virulence and resistance factors, including gene products associated with Adherence (*n*=50–54), Cellular metabolism and nutrient uptake (*n*=10), Damage (*n*=6–7), Invasion and intracellular survival (*n*=33–36), Resistance to antibiotics and toxic compounds (*n*=65–81), and Other (*n*=16–18) (Data S3). In general, virulence factors and antimicrobial/toxin resistance factors were almost identical in number among the Vietnamese strains and were comparable to non-Vietnamese strains. However, some genes were absent in most Vietnamese strains but present in most non-Vietnamese strains, such as ‘MCE-family protein Mce1D’, ‘MCE-family protein Mce1F’, ‘Chromate transport protein ChrA’, ‘NAD(P)H oxidoreductase YRKL (EC 1.6.99.-) Putative NADPH-quinone reductase (modulator of drug activity B) Flavodoxin 2’ and ‘Tellurite resistance protein TerB’. In contrast, ‘Hemolysins and related proteins containing cystathionine-β-synthase domains’ was found only in EM150506. Several experimentally verified virulence factors identified in *N. seriolae* and other *Nocardia* species, including catalase, superoxide dismutase, phospholipase C and protease [63], were present in all Vietnamese and non-Vietnamese strains, indicating that they are highly conserved genes within this genus.

## Discussion

PFGE has conventionally been considered the ‘gold standard’ for studying the genetic diversity of many different pathogenic bacteria species, including *N. seriolae* [19, 20, 30, 64]. PFGE has previously identified multiple pulsotypes among isolates retrieved from fish in Japan and Taiwan [19, 20]. Notably, one study identified identical pulsotypes between certain Taiwanese 1997–2007 outbreak strains and Japanese *N. seriolae* isolated from yellowtail in 2002 (pulsotypes X1 and A1) and 2005 (pulsotype X11) [20], suggesting at least two transmission events between Taiwan and Japan. Unlike *N. seriolae* from Japan and Taiwan, all 20 Vietnamese isolates fell into a single cluster, even when using a combination of *Xba*I and *Ase*I. However, PFGE lacked the resolution to differentiate Vietnamese isolates into the two clades identified using phylogenomic analysis. This limited resolution has also been documented for other bacteria such as *Salmonella enterica* [65]*, Listeria monocytogenes* [66] and *Escherichia coli* [67]. It was unfortunately not practical to compare the Vietnamese pulsotypes with published studies due to known challenges with interlaboratory standardization using PFGE [68]; therefore, it is not known whether the Vietnamese PFGE cluster has been previously reported.

Next-generation sequencing provides excellent resolution, accuracy and data portability, and as such, has begun replacing PFGE as the new gold standard for nocardiosis outbreak analyses [69]. To illustrate the value of WGS for nocardiosis epidemiological investigations, we sequenced seven representative Vietnamese *N. seriolae* strains and compared them with all publicly available genomes (*n*=13). Like PFGE, the limited genomic variation (0–5 SNPs; Fig. 3) observed among Vietnamese strains confirms a recent, single introduction into Vietnam, with subsequent dissemination across multiple mariculture facilities within the South Central Coast region. Phylogenomic analysis showed that Vietnamese strains were most closely related to UTF1, which was isolated from farmed yellowtail in Japan in 2008 [27]; this strain differed from the Vietnamese common ancestor by just 220 SNPs (MRCA: ~1998). Shimahara and colleagues [20] have previously postulated that transboundary translocation of live fish stocks asymptomatically infected with *N. seriolae* from China and Hong Kong may have introduced new strains into Japan. Wild-caught amberjack juveniles, one of the most susceptible host species for *N. seriolae* infection, was also reportedly imported into Japan from Vietnam in 2000 [70]. However, there has not yet been a case of nocardiosis reported in Vietnam in other aquatic species besides *Trachinotus* species, and the first of these cases were only recorded in 2012 [9]; therefore, it is unlikely that the Japanese *N. seriolae* was introduced from amberjack imported from Vietnam. Based on our genomic analysis, it is plausible that *N. seriolae* from Japan has been introduced into other countries such as Vietnam given that international export of valuable aquaculture fish species is relatively common; however, there is a paucity of information about import–export of live fish stocks from Japan or Vietnam, and, as such, this hypothesis cannot be confirmed.

Our BEAST results (Fig. S3) add further to our hypothesis of a recent introduction of *N. seriolae* into Vietnam from infected *Trachinotus* species. Our analysis showed that *N. seriolae* introduction into Vietnam occurred in ~2001 (95 % HPD: 1999–2003), which fits with the Taiwanese/Japanese outbreaks occurring in the late 1990s and early 2000s. We unfortunately lack isolate data from Taiwan that could suggest the directionality of transfer, or that could provide more accurate source attribution; nevertheless, we have been able to make some interesting and useful insights into the evolutionary history of *N. seriolae* in Vietnam based on this dated phylogeny.

Whilst our results suggest a probable Asian origin for the Vietnamese outbreaks, there are few publicly available *N. seriolae* genomes (only 20 as of 11 February 2022, including seven from our study), and none from other Asian regions such as Taiwan [20], Singapore, Malaysia, or Indonesia [71], or non-Asian regions such as Mexico [23] and USA [21] where *N. seriolae* outbreaks have been documented; therefore, the precise origin of the Vietnamese outbreaks and mode of *N. seriolae* introduction currently remain unresolved. Concerningly, our results, and those of others, demonstrate that, unchecked, *N. seriolae* transmission may represent a substantial unmitigated risk to fish aquaculture. It is thus an utmost imperative to establish domestic and international monitoring processes for *N. seriolae* for both farmed and wild species, including the implementation of molecular methods to characterize new outbreaks, to prevent the spread of this devastating pathogen into new environments, and associated heavy economic losses and food security concerns.

To facilitate the rapid identification of *N. seriolae* genotypes among our Vietnamese strains, we designed inexpensive SYBR-MAMAs targeting two phylogenetically informative SNPs. The first SNP assay robustly differentiates Vietnam from non-Vietnamese strains, thereby permitting prospective identification of newly transmitted strains into Vietnam, an essential facet in future fish importation biocontrol efforts. This assay can also be used to monitor for the emergence of Vietnamese strains in new regions, such as new aquaculture facilities in Vietnam, or prior to export of fingerlings to other countries. The second SNP assay rapidly differentiates strains belonging to the two Vietnamese clades. By applying this second assay to the 20 Vietnamese strains, we observed that both clades were well disseminated across all four provinces: Khánh Hòa, Ninh Thuận, Phú Yên and Vũng Tàu. Phylogenomic analysis of seven representative Vietnamese strains also showed dispersal of these two clades among three of the four provinces. Although unconfirmed, it is probable that the widespread trade of eggs, fingerlings and live permit fish for aquaculture in Vietnam since industry inception in the early 2000s, including local unmonitored trade among fish farmers, has driven the successful dissemination of *N. seriolae* among Vietnamese permit farms. Taken together, our findings highlight the large risk of undetected *N. seriolae* dispersal among mariculture facilities and the need for establishing strict monitoring practices to prevent further pathogen transmission.

WGS is currently laborious, expensive and inaccessible to most laboratories in Vietnam and many other Asian countries. Using comparative genomics, we established a catalogue of SNPs specific to each clade and subclade. This SNP database may be useful for both targeted resequencing efforts and the design of phylogenetically robust genotyping methods to permit source tracing of future *N. seriolae* outbreaks without the requirement for further WGS or bioinformatic analyses. The SYBR-MAMAs developed in this study successfully detected two phylogenetically informative SNPs, with genotyping results fully concordant with WGS, confirming that SYBR-MAMA is a valuable and inexpensive diagnostic method for SNP characterization.

Very little is known about the pathogenesis of *Nocardia* species, which are capable of invading host macrophages and preventing the fusion of phagosomes with lysosomes, leading to long-term survival and proliferation in host cells [72]. Due to the paucity of available genomic data for this pathogen, a final aspect of this study was to better understand virulence and antimicrobial resistance factors encoded by the *N. seriolae* genome. Our analysis of 2020 *N. seriolae* genomes is the largest genomic assessment of this pathogen to date, and largely corroborates the conclusions drawn from a previous analysis of seven *N. seriolae* genomes, which showed that *N. seriolae* have >99.9 % Orthologous Average Nucleotide Identity values [28]. Analysis of the genome content of seven Vietnamese *N. seriolae* strains revealed that, like non-Vietnamese strains, they encode a high proportion of ‘hypothetical protein’ genes (i.e. 45.8 %), a finding that highlights the need for more studies to investigate the functions of these genes. More than 180 core genes (present in all strains) were found to code for antimicrobial resistance and virulence factors in the Vietnamese strains, including genes associated with Adherence (*n*=49), Cellular metabolism and nutrient uptake (*n*=10), Damage (*n*=6), Invasion and intracellular survival (*n*=33), Resistance to antibiotics and toxic compounds (*n*=26), and Others (*n*=11) that may possibly account for the main virulence traits of this fish pathogen. The presence of conserved genes encoding β-lactamase class C-like and penicillin-binding proteins (*n*=11), multidrug resistance protein ErmB (*n*=1), probable multidrug resistance protein NorM (*n*=1) and a small multidrug resistance family protein (*n*=1) in all *N. seriolae* genomes may explain observed antimicrobial resistance towards penicillin and cephalexin, two β-lactam antibiotics that are commonly used to treat nocardiosis in Vietnamese permit fish farms (data not shown).

CRISPRs, which are encoded by many bacterial and archaeal species, defend against invasive mobile genetic elements such as viral or plasmid DNA [73], and also play a role in bacterial pathogenesis, biofilm formation, adherence, programmed cell death and quorum sensing [74]. Acquisition and maintenance of CRISPR-Cas systems are greatly influenced by environmental conditions and microbial communities [75]. Recent research has shown that 40 % of CRISPR-Cas loci are away from any associated *cas* genes or are not associated with *cas* genes, which are known as orphan CRISPR arrays [76]. Like many other bacterial species such as *Listeria monocytogenes *, *Aggregatibacter actinomycetemcomitans *, *Enterococcus faecalis *, *Staphylococcus* spp., *Pseudomonas aeruginosa* and *Salmonella enterica* [77–81], orphan CRISPR arrays were found in *N. seriolae* genomes. These incomplete CRISPR-Cas systems may be a remnant of decaying loci that are recruited and*/*or selectively maintained to perform important, but as yet unknown, biological functions [73]. Alternatively, our results may be an artefact of current CRISPR-Cas prediction tools, which predict the CRISPRs primarily based on the typical CRISPR structure [77]. As the role of these CRISPR loci in *N. seriolae* is not yet known, further work is needed to uncover their precise role in this pathogen.

In conclusion, our study provides novel insights into the epidemiology of *N. seriolae* outbreaks in farmed permit fish in Vietnam. Our detailed molecular and genomic analyses revealed minimal genomic diversity among Vietnamese *N. seriolae* isolates. Unlike PFGE, WGS detected strain variation at single-base resolution, and identified two distinct Vietnamese clades that share recent ancestry. Our results indicate recent importation of a single *N. seriolae* clone into Vietnam, which has then led to a nationwide outbreak of nocardiosis in permit fish farms. The analysis of additional genomes, particularly from other geographical regions, will be important for better understanding *N. seriolae* evolution, and will enable more precise investigations into the origin and transmission of this devastating pathogen. Finally, our SNP assays provide a rapid and inexpensive method for genotyping of ongoing and future nocardiosis outbreaks in Vietnam.

## Supplementary Data

Supplementary material 1
