# Supplementary material for: Comparative genomics of Nocardia seriolae reveals recent importation and subsequent widespread dissemination in mariculture farms in the South Central Coast region, Vietnam
Source: Microb Genom. 2022 Jul 4;8(7):mgen000845. doi: 10.1099/mgen.0.000845 (PMC9455698; doi:10.1099/mgen.0.000845)
Supplement: Supplementary material 1 [file mgen-8-845-s001.pdf]

Supplemental Figures for Le *et al*., 2022. **Comparative genomics of *Nocardia seriolae* reveals recent importation and subsequent widespread dissemination in mariculture farms in South Central Coast, Vietnam.** *Microb Genom*.

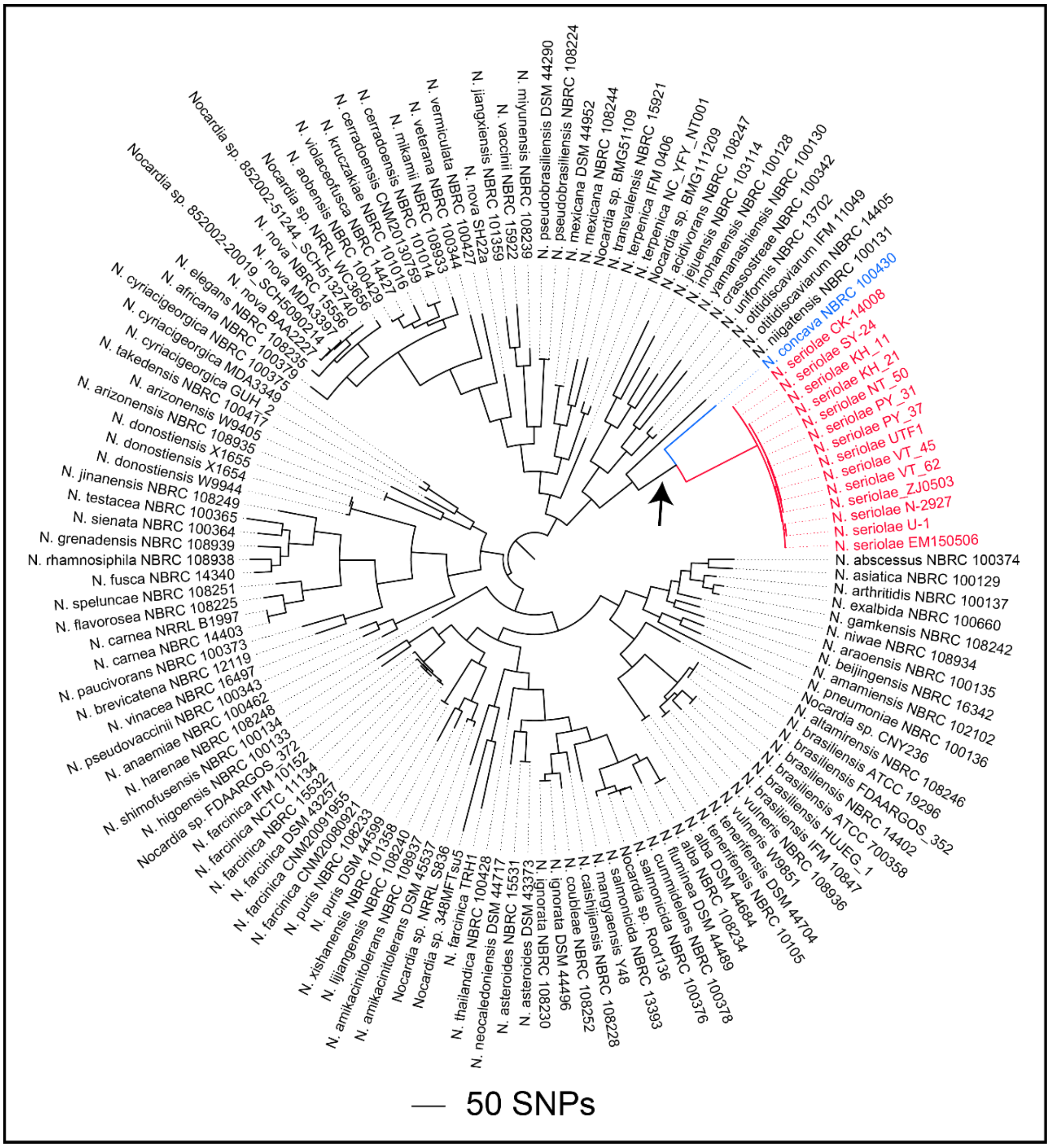

Fig. S1. Maximum parsimony phylogenetic analysis of 134 *Nocardia* genomes representing 78 assigned species and 10 strains of unassigned species. The branch showing *N. seriolae* (red text) and its nearest neighbour, *N. concava* (blue text), is denoted by a black arrow. Consistency index=0.12.

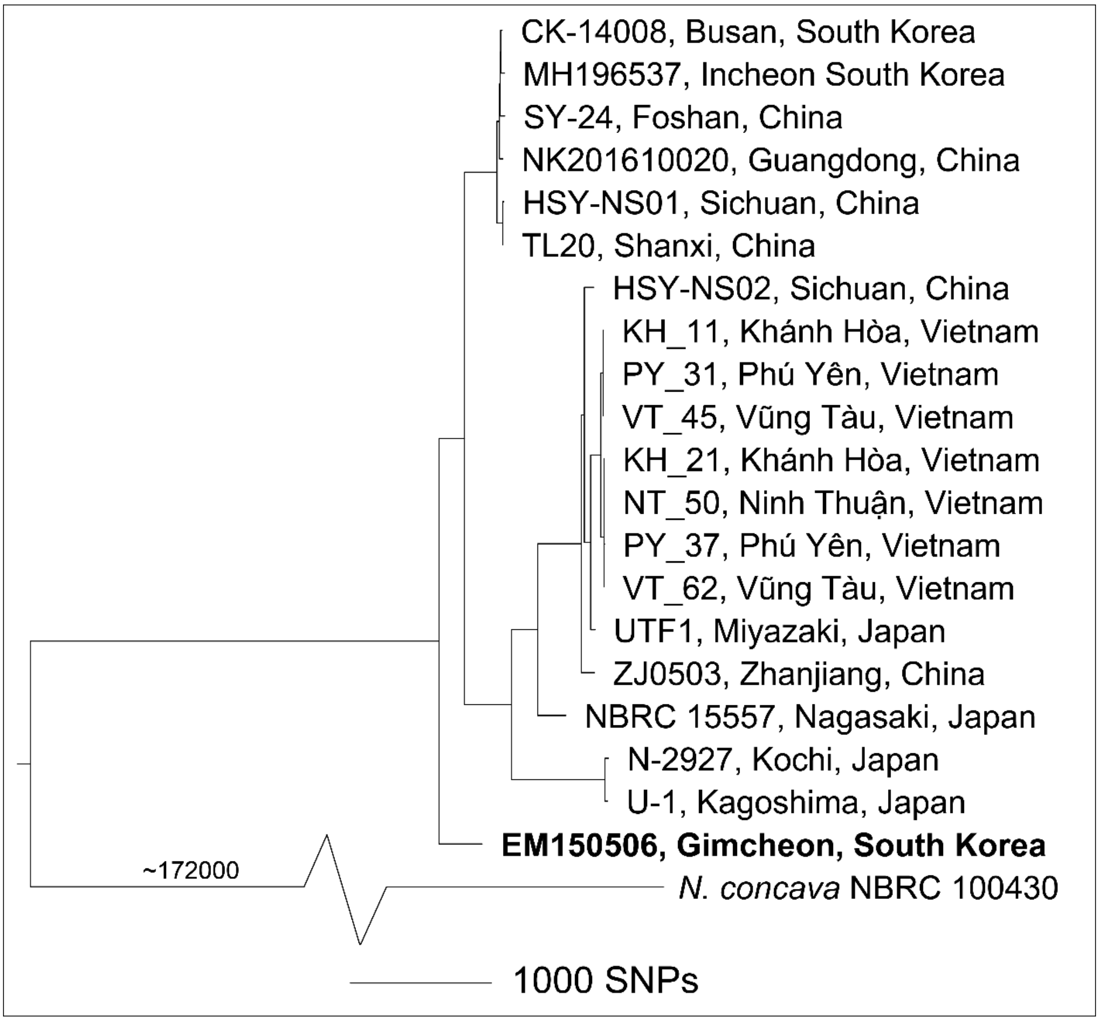

Fig. S2. Maximum parsimony phylogenetic analysis of 20 *Nocardia seriolae* strains, rooted with the nearest neighbour species, *N. concava* (according to **Fig. S1**). A total of 175,177 high-confidence, biallelic, orthologous, core-genome single-nucleotide polymorphisms were used to construct the phylogeny, with the long branch length to *N. concava* shortened to enable visualisation of genetic variation among the *N. seriolae* strains. This analysis demonstrates that South Korean strain EM150506 (bolded) is the most ancestral *N. seriolae* strain; EM150506 was used to root the *N. seriolae*-only tree (**Fig. 3**). This information was used to root the within-species phylogeny (**Fig. 3**). Consistency index=0.998.

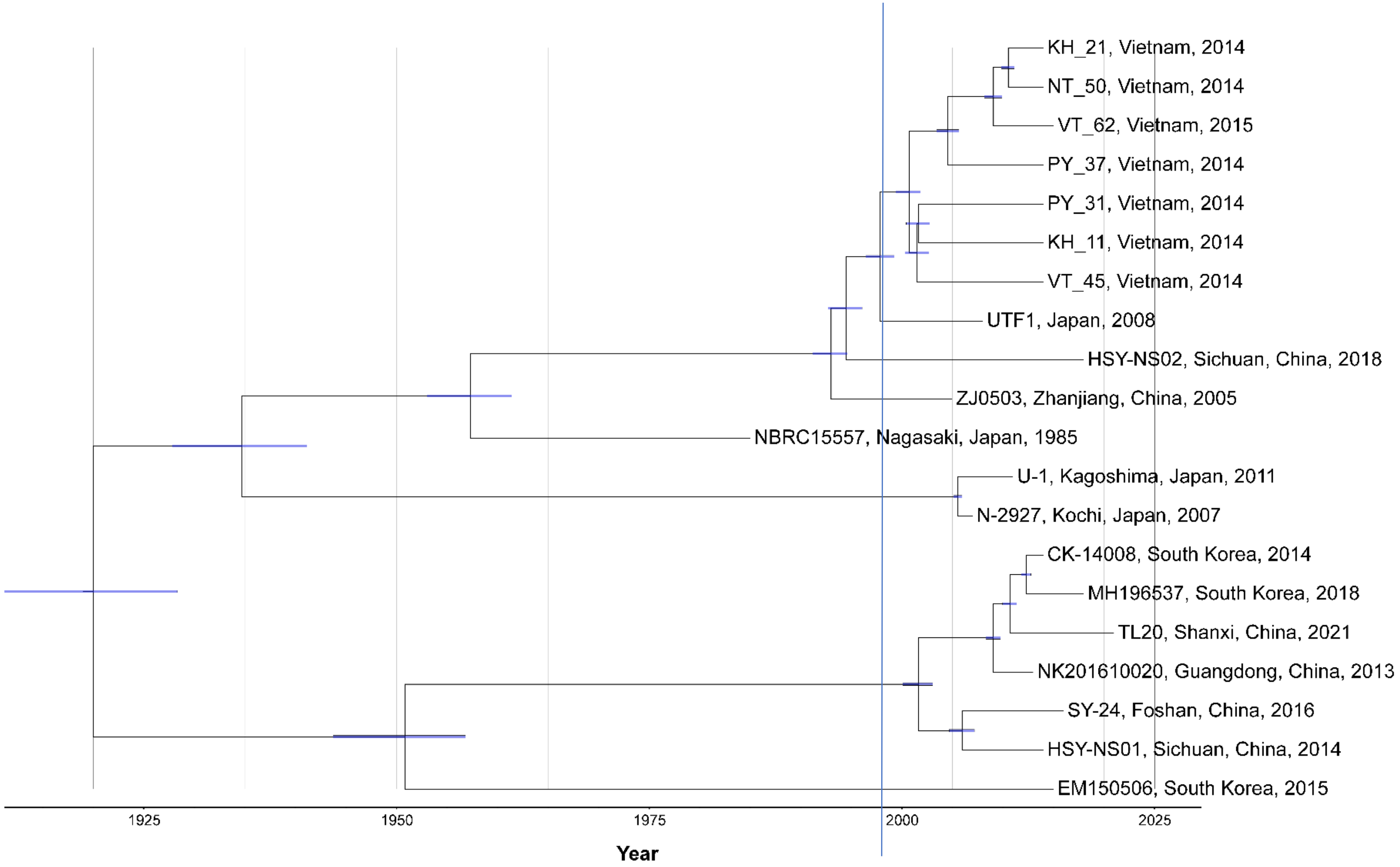

Fig. S3. Bayesian phylogenetic analysis of all 20 *Nocardia seriolae* genomes, including the seven new *N. seriolae* genomes from Vietnam that were generated in this study. Blue bars indicate 95% highest posterior density.
